# H19 may regulate the immune cell infiltration in carcinogenesis of gastric cancer through miR-378a-5p/SERPINH1 signaling

**DOI:** 10.1186/s12957-022-02760-6

**Published:** 2022-09-14

**Authors:** Jianxin Li, Ting Han, Xin Wang, Yinchun Wang, Xuan Chen, Wangsheng Chen, Qingqiang Yang

**Affiliations:** Department of General Surgery (Gastrointestinal Surgery), The Affiliated Hospital of Southwest Medical University, Luzhou, Sichuan 646000 People’s Republic of China

**Keywords:** Gastric cancer, Noncoding RNA, Competing endogenous RNA, Biomarker, Immune cell infiltration

## Abstract

**Background:**

Increasing studies have indicated that noncoding RNA (ncRNA)-mediated competing endogenous RNA (ceRNA) network serves as a significant role in cancer progression, but the underlying regulatory mechanisms of which in gastric cancer (GC) remain largely unclear.

**Methods:**

Based on Gene Expression Omnibus and The Cancer Genome Atlas datasets, potential biomarkers for GC were screened and validated by machine learning. Then, upstream regulatory ncRNA of potential biomarkers was identified to construct a novel ceRNA network in GC through means of stepwise reverse prediction and validation. Ultimately, tumor immune cell infiltration analysis was performed based on the EPIC algorithm.

**Results:**

A total of 188 differentially expressed genes (DEGs) were screened, and three candidate diagnostic biomarkers (FAP, PSAPL1, and SERPINH1) for GC were identified and validated. Subsequently, H19 and miR-378a-5p were identified as upstream regulatory ncRNAs that could potentially bind SERPINH1 in GC. Moreover, Immune infiltration analysis revealed that each component in the ceRNA network (H19/miR-378a-5p/SERPINH1) was significantly correlated with the infiltration abundances of diverse tumor-infiltrating immune cells.

**Conclusions:**

H19 may regulate the immune cell infiltration in carcinogenesis of GC through miR-378a-5p/SERPINH1 signaling.

**Supplementary Information:**

The online version contains supplementary material available at 10.1186/s12957-022-02760-6.

## Introduction

Gastric cancer (GC) is one of the most commonly diagnosed malignant tumors in the digestive system, which accounts for the third leading cause of cancer-related deaths despite its worldwide decline in incidence and mortality over the past five decades [1, 2]. Although numerous efforts have been afforded to determine the pathogenesis of GC and substantial improvement in diagnosis and therapy has been achieved, the prognosis of GC patients is still comparatively poor [3]. The majority of GC patients are diagnosed in the middle to late stage and therefore lose the opportunity to be cured [4]. Therefore, it is imperative to explore the regulatory mechanisms of GC, which not only contributes to improving the understanding of the pathogenesis of GC but also provides novel biomarkers for the diagnosis and therapy of GC.

It is widely accepted that noncoding RNA (ncRNA) could regulate the progression of multiple diseases through modulating gene expression at the transcriptional and posttranscriptional levels [5]. In 2011, Salmena et al. proposed the competing endogenous RNA (ceRNA) hypothesis that long noncoding RNA (lncRNA) can suppress mRNA degradation or silence mRNA translation by sponging microRNA (miRNA), thereby affecting protein coding and modulating the disease process [6]. Recently, increasing studies have demonstrated that the ceRNA network might play critical roles in initiation and progression of multiple diseases, including cancer. For example, Xu et al. reported that lncRNA SNHG1 exerted as a sponge for miR-154-5p, thereby facilitating colorectal cancer cell growth through activating the downstream target of miR-154-5p, CCND2 [7]. Xin et al. demonstrated that lncRNA LINC01133 accelerates proliferation and aggressive of liver cancer cells by sponging miR-199a-5p to activate the ANXA2/STAT3 signaling pathway [8]. Zhao et al. indicated that lncRNA HOTAIR promotes cell growth, metastasis, and apoptosis of breast cancer through the miR-20a-5p/HMGA2 signaling [9]. Moreover, several studies have also reported that ceRNA network could play a vital role in the carcinogenesis of GC [10]. Nevertheless, pivotal lncRNA-miRNA-mRNA ceRNA networks involved in the progression of GC still need to be clarified.

In the present study, we first identified a list of diagnostic biomarkers closely related to GC from Gene Expression Omnibus dataset by machine learning and validated them in The Cancer Genome Atlas dataset. Then, by using multiple bioinformatic methods, the upstream regulatory miRNA and lncRNA were reversely predicted and validated from the perspectives of expression pattern and prognostic value. Ultimately, a novel ceRNA regulatory network was successfully developed, and each component in the network utterly conformed with ceRNA theory and meanwhile associated with the prognosis of GC patients.

## Materials and methods

### Data collection and processing

Gene expression microarray data sets GSE13911, GSE19826, and GSE79973 were downloaded from the Gene Expression Omnibus (GEO, http://www.ncbi.nlm.nih.gov/geo/). Among them are as follows:

- GSE13911 including 31 normal samples and 38 GC samples [11]
- GSE19826 including 15 normal samples and 12 GC samples [12]
- GSE79973 including 10 normal samples and 10 GC samples [13]

All of these three datasets were based on GPL570 platform [HG-U133_Plus_2] Affymetrix Human Genome U133 Plus 2.0 Array. Then, these three datasets were merged into a combined dataset and used as the training cohort, and the surrogate variable analysis (SVA) algorithm was applied to eliminate the batch effect between any two datasets [14]. Besides, the RNA-sequencing data of GC was downloaded from The Cancer Genome Atlas (TCGA, https://cancergenome.nih.gov/) and used as the testing cohort.

### Differentially expressed gene screening

Differentially expressed genes (DEGs) between normal samples and GC samples were analyzed by using the “limma” package in R in the training cohort [15]. The cutoff criteria for identifying DEGs was as follows: | log2-fold change (FC) | ≥ 2 and adjusted *P* < 0.05.

### Biological function enrichment analysis

Gene ontology (GO) analysis is commonly used to annotate the biological functions or localization of genes from the perspective of biological processes (BP), cellular components (CC), and molecular functions (MF) [16]. Kyoto Encyclopedia of Genes and Genomes (KEGG) pathway enrichment analysis database is a knowledge resource for systematic analysis of gene functions, linking genomic information with signaling pathways [17]. Disease ontology (DO) analysis is usually applied to assess how particular genes may be involved in or influenced by specific disease states [18]. In the present study, we performed GO, KEGG, and DO analyses by using the “clusterProfiler” R package to analyze the DEGs at the functional level [19]. Only the enriched terms with *P* < 0.05 were considered statistically significant.

### Diagnostic gene identification and verification

The least absolute shrinkage and selection operator (LASSO) is a regression-based algorithm that has the unique feature of penalizing the absolute value of a regression coefficient, thus automatically avoiding the overfitting and removing uninfluential variates [20]. In the present study, we created LASSO logistic regression model using the “glmnet” package to screen the potential diagnostic biomarkers from DEGs for GC in the training cohort. In addition, support vector machine recursive feature elimination (SVM-RFE), an algorithm widely used for cancer classification, biomarker discovery, and cancer driver gene discovery, was performed to further identify these biomarkers with diagnostic value in GC through the “e1071” package [21]. Area under receiver operating characteristic curve (AUC) was calculated to assess the predictive accuracy of the candidate diagnostic biomarkers. Furthermore, we also evaluated the diagnostic and prognostic values of the candidate diagnostic biomarkers in TCGA cohort.

### Identification of upstream miRNA

The upstream miRNAs that interacted with candidate diagnostic genes were screened by using miRTarBase (http://mirtarbase.mbc.nctu.edu.tw/index.html), an online platform whose miRNA-target interactions have been validated using distinct types of experiments, including reporter assay, Western blot, microarray, and next-generation sequencing technologies [22]. Then, we performed correlation analysis, differential analysis, and survival analysis on these upstream miRNAs to filter out candidate miRNAs that could potentially bind to candidate diagnostic genes based on TCGA project. The criteria for candidate miRNAs were defined as follows: (1) negatively correlated with its targeted mRNA, (2) differentially expressed between GC and normal samples, and (3) correlated with the prognosis of patients with GC. A *P*-value < 0.05 was considered statistically significant.

### Identification of upstream lncRNA

The upstream lncRNAs that sponged with candidate miRNA were identified by exploring LncBase v2 (www.microrna.gr/LncBase), a reference repository that contains an extensive collection of miRNA-lncRNA interactions which has been experimentally validated [23]. Similarly, we also performed correlation analysis, differential analysis, and survival analysis to screen out candidate lncRNAs based on TCGA project. The criteria were defined as follows: (1) negatively correlated with its interacted miRNA and meanwhile positively correlated with downstream mRNA, (2) differentially expressed between GC and normal samples, and (3) correlated with the prognosis of patients with GC. *P*-value < 0.05 was considered statistically significant.

### Profile of immune cell infiltration

To determine the correlation between the proportion and composition of tumor-infiltrating immune cells (TIICs) and the expression of candidate diagnostic genes, we applied the EPIC (http://epic.gfellerlab.org) algorithm to assess the abundances of TIICs among the GC samples from the TCGA project [24]. Then, GC samples were divided into low- and high-expression groups according to the median expression level of mRNA, miRNA, and lncRNA, and the differences in TIIC content between the low- and high-expression groups were analyzed. In addition, we further investigated the prognostic value of distinct TIICs in GC patients.

### Statistical analysis

All statistical analysis was performed using the R software (v4.1.1 https://www.r-project.org/). Differential expression analysis was assessed by Wilcoxon signed-rank test. Fisher’s test was applied to screen the significant GO, KEGG, and DO enrichment terms. Correlation analysis was estimated by Spearman correlation coefficients. The log-rank test was used in the Kaplan-Meier survival curve analysis. A *P*-value < 0.05 was considered statistically significant.

## Results

### Identification of differentially expressed genes

**Fig. 1:**
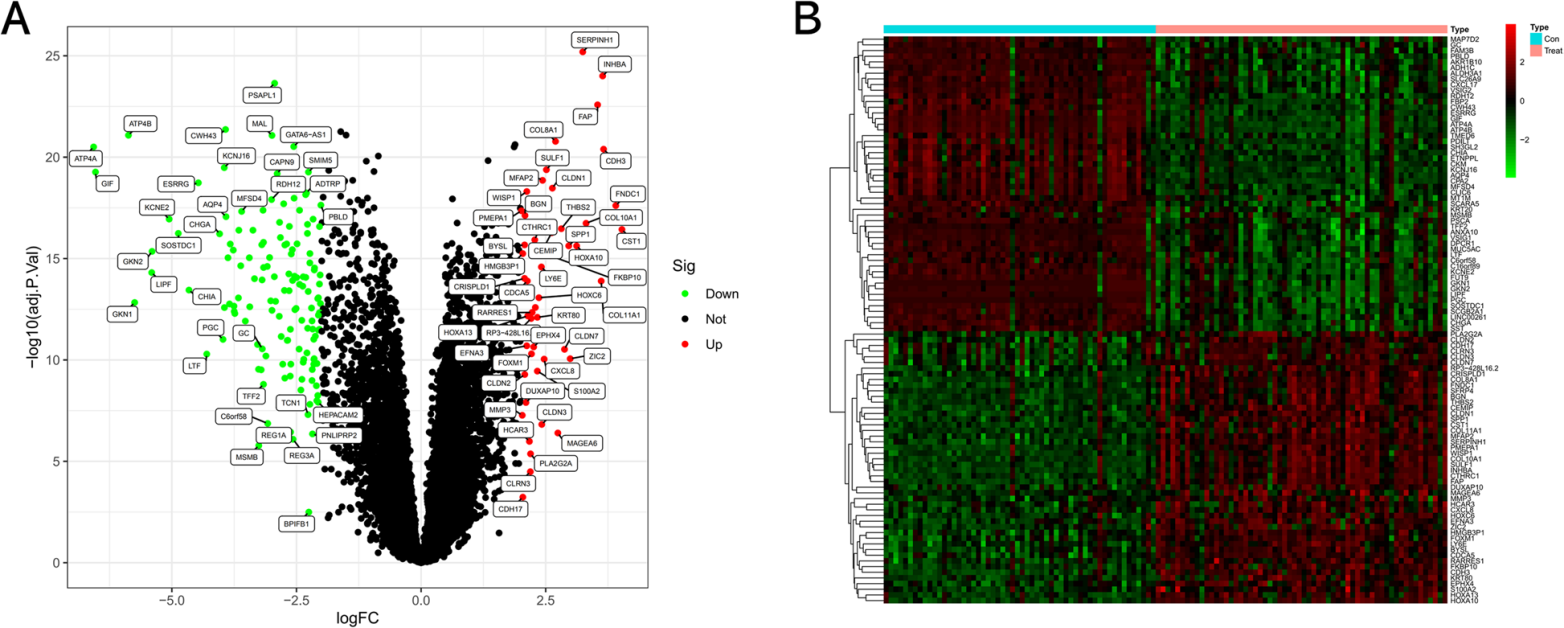
Differential expression analysis. **A** Volcano plot between GC and control groups. **B** Heat map of DEGs between GC and controls

First, a combined dataset including 56 normal samples and 60 GC samples was generated, and its gene expression matrix was normalized, and the batch effects were removed. Subsequently, a total of 188 DEGs between normal samples and GC samples were identified, including 48 upregulated DEGs and 140 downregulated DEGs (Supplementary Table 1). The volcano plot of these DEGs was displayed in Fig. 1A, and the expression heat map was presented in Fig. 1B.

### Function enrichment analysis

**Fig. 2:**
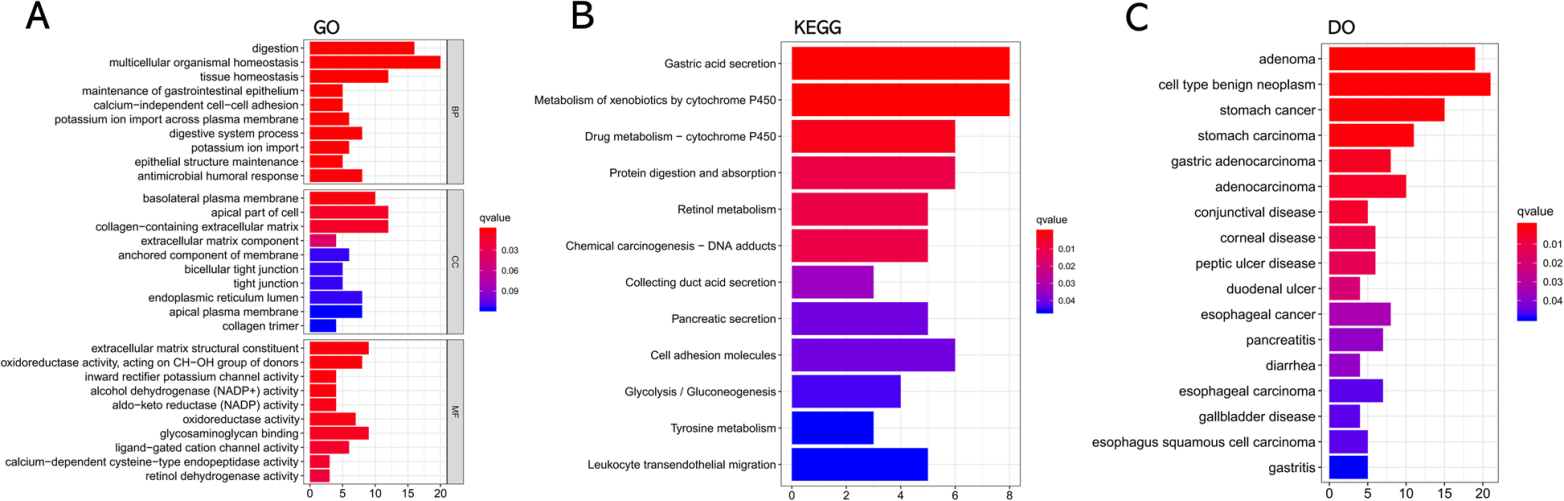
Enrichment analysis. **A** GO analysis of DEGs. **B** KEGG pathways enrichment analysis of DEGs. **C** DO enrichment analysis of DEGs

The GO annotation analysis found that these DEGs participated in biological process of digestion, tissue homeostasis, and maintenance of gastrointestinal epithelium. Cellular component enriched these DEGs mainly in basolateral plasma membrane, apical part of cell, and collagen-containing extracellular matrix. Besides, molecular function suggested enrichment mainly at extracellular matrix structural constituent, oxidoreductase activity, and glycosaminoglycan binding (Fig. 2A). KEGG enrichment analysis revealed that these DEGs mainly enriched in gastric acid secretion, metabolism of xenobiotics by cytochrome P450, drug metabolism-cytochrome P450, and protein digestion and absorption (Fig. 2B). Moreover, DO enrichment found that these DEGs mainly involved in adenoma, cell type benign neoplasm, and stomach cancer (Fig. 2C).

### Identification of candidate biomarkers in GC

**Fig. 3:**
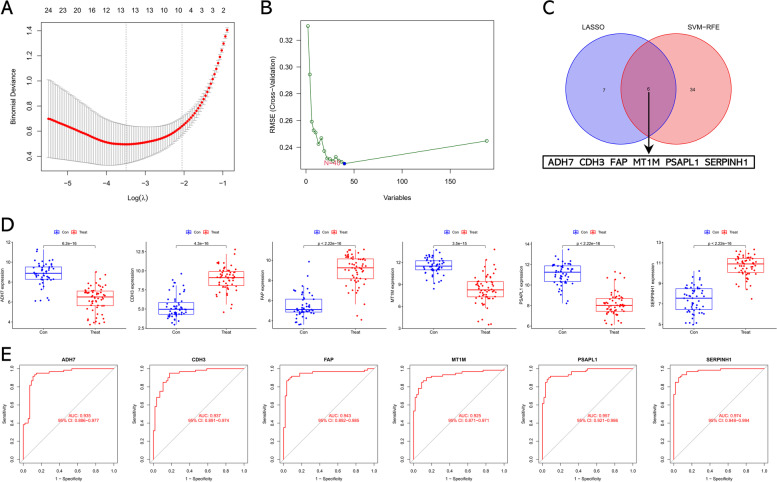
Key genes were identified by LASSO and SVM-RFE. **A** Key genes identified by LASSO algorithm. **B** Key genes identified by SVM-RFE algorithm. **C** Venn diagram showing the intersection of candidate biomarkers between LASSO and the SVM algorithm. **D** Box plots of candidate biomarkers (ADH7, CDH3, FAP, MT1M, PSAPL1, and SERPINH1) expression between GC samples and normal samples. **E** ROC analysis of ADH7, CDH3, FAP, MT1M, PSAPL1, and SERPINH1 was performed on the training cohort

We applied LASSO and SVM-RFE algorithms to identify candidate biomarkers among these DEGs. As a result, 13 DEGs were identified as potential biomarkers of GC according to the LASSO algorithm, while 40 DEGs were potential biomarkers of GC based on the SVM-RFE method (Fig. 3 A & B). Finally, six overlapped candidate biomarkers were identified by using the Venn diagram, including ADH7, CDH3, FAP, MT1M, PSAPL1, and SERPINH1 (Fig. 3C). The expression pattern of these potential biomarkers for GC in the training cohort was presented in Fig. 3D. In addition, we further examined the diagnostic efficiency of these potential biomarkers through ROC curves in the training cohort. As shown in Fig. 3E, the results found that the AUC of all these potential biomarkers was higher than 0.9, suggesting that ADH7, CDH3, FAP, MT1M, PSAPL1, and SERPINH1 are effective indicators of GC.

### Validation of candidate biomarkers in GC based on TCGA

**Fig. 4:**
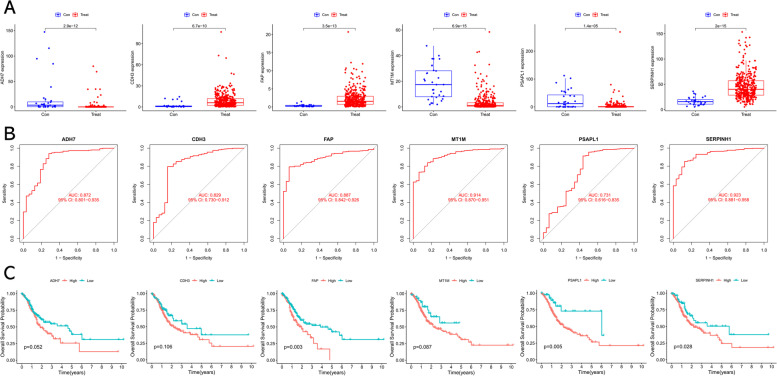
Validation of key genes in TCGA project. **A** Box plots of candidate biomarkers (ADH7, CDH3, FAP, MT1M, PSAPL1, and SERPINH1) expression between GC samples and normal samples based on TCGA cohort. **B** ROC analysis of ADH7, CDH3, FAP, MT1M, PSAPL1, and SERPINH1 was performed on the TCGA cohort. **C** Kaplan-Meier survival curves of candidate biomarkers (ADH7, CDH3, FAP, MT1M, PSAPL1, and SERPINH1) in GC patients based on TCGA cohort

To enhance the reliability of our findings, the diagnostic efficiency of these potential biomarkers was further validated in the testing cohort based on TCGA project. First, RNA-sequencing data of 32 normal samples and 375 GC samples, and corresponding clinical information, were obtained from TCGA. Then, differential analysis revealed that the expression pattern of these five potential biomarkers was coincident with the results in the training cohort (Fig. 4A). ROC curves showed that the AUC of ADH7, CDH3, FAP, MT1M, PSAPL1, and SERPINH1 were 0.872, 0.829, 0.887, 0.914, 0.731, and 0.923, respectively (Fig. 4B). Finally, we further performed survival analysis to evaluate the prognostic value of these potential biomarkers and found that dysregulation of FAP, PSAPL1, and SERPINH1 was significantly correlated with the prognosis of GC patients (Fig. 4C). Thus, FAP, PSAPL1, and SERPINH1 were identified as candidate biomarkers in GC and selected for subsequent study.

### Identification of candidate upstream miRNA

**Fig. 5:**
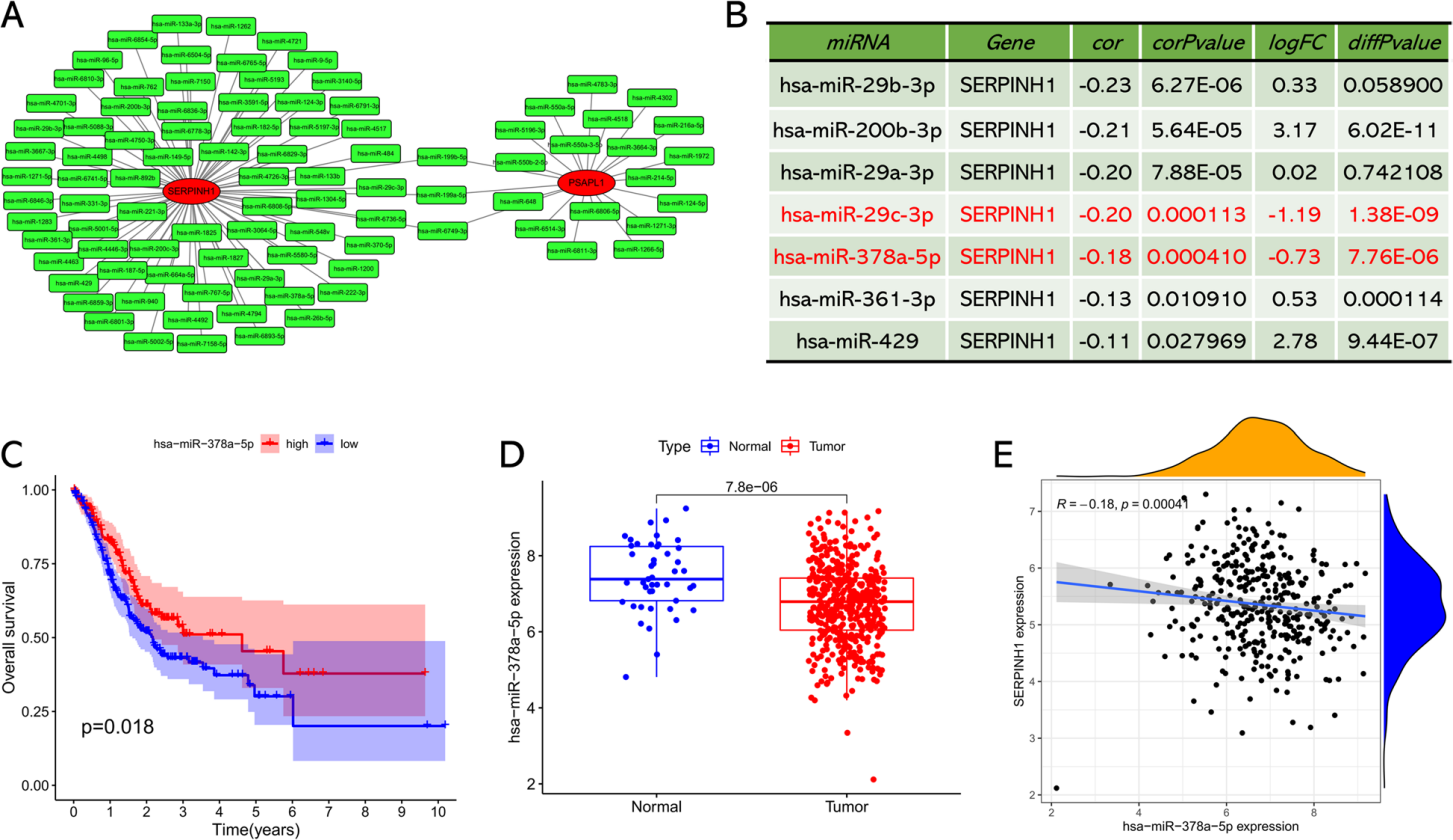
Identification of miR-378a-5p as a potential upstream miRNA of SERPINH1 in GC. **A** The miRNA-SERPINH1/PSAPL1 regulatory network constructed by Cytoscape software. **B** The expression correlation between predicted miRNAs and SERPINH1 in GC. **C** The prognostic value of miR-378a-5p in GC assessed by Kaplan-Meier plotter. **D** The expression boxplot of miR-378a-5p in GC and normal samples determined by TCGA database. **E** The expression pattern between miR-378a-5p and SERPINH1 assessed by Spearman correlation

We conducted miRTarBase database to predict the upstream miRNA of candidate biomarkers and found that 94 miRNAs interacted with PSAPL1 and SERPINH1, while FAP was not observed to interact with any miRNA in this database. Then, a miRNA-mRNA network comprised of 97 miRNA-mRNA relationship pairs was constructed using Cytoscape (http://cytoscape.org/) (Fig. 5A) [25]. According to the inverse regulatory relationship between mRNA and miRNA, we further performed correlation analysis and differential analysis on these upstream miRNAs. As shown in Fig. 5B, seven upstream miRNAs showed a negative correlation with SERPINH1, and only two of which (hsa-miR-29c-3p and hsa-miR-378a-5p) were downregulated in GC. As for PSAPL1, there was no upstream miRNA that showed a negative correlation with it. Subsequent survival analysis revealed that only hsa-miR-378a-5p was significantly associated with a favorable prognosis in GC patients (Fig. 5C). Thus, hsa-miR-378a-5p was identified as a candidate miRNA that could be the most potential regulatory miRNA of SERPINH1 in GC and chosen for further analysis. The expression boxplot of hsa-miR-378a-5p in GC was presented in Fig. 5D, and the correlation between hsa-miR-378a-5p and SERPINH1 was presented in Fig. 5E.

### Identification of candidate upstream lncRNA

**Fig. 6:**
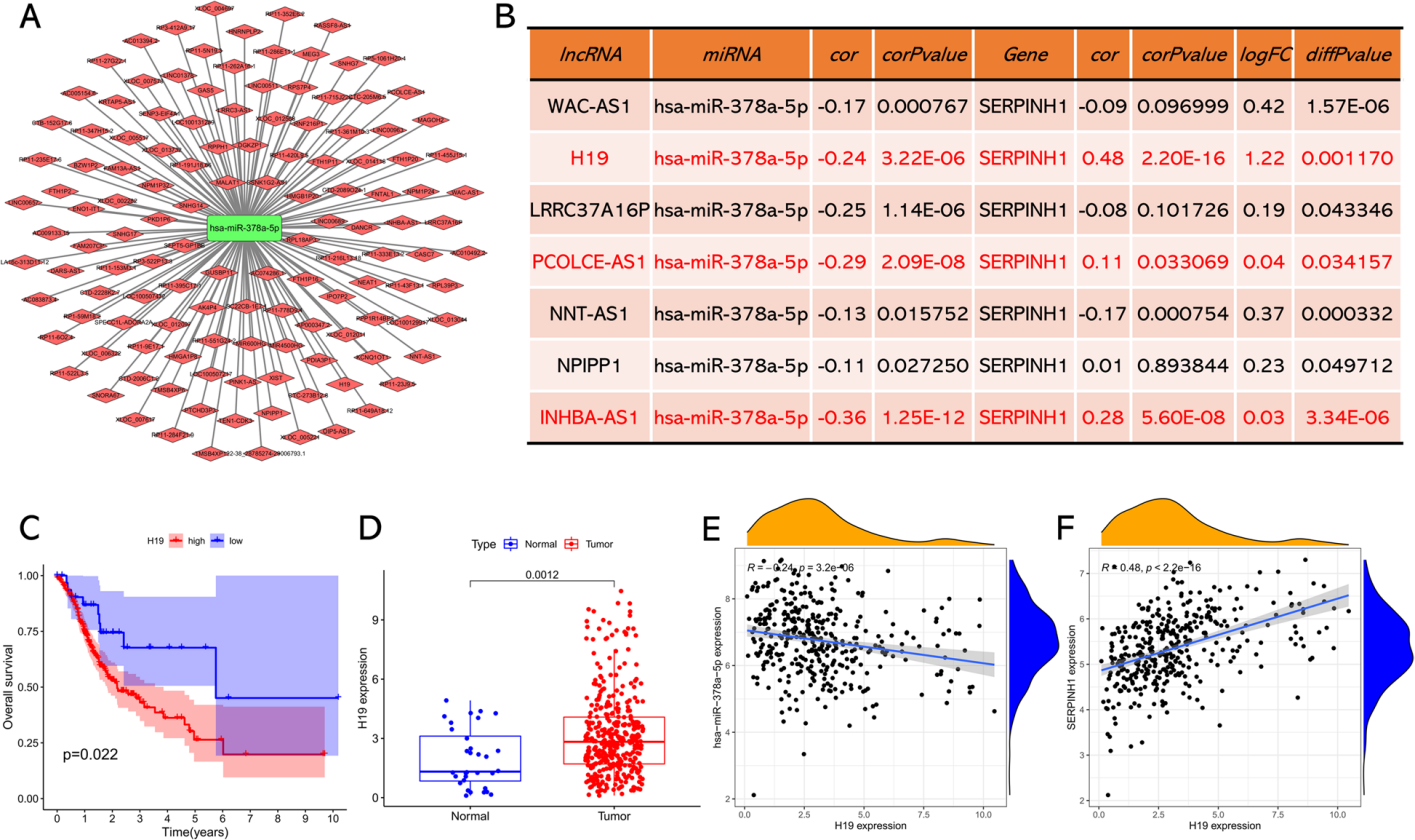
Identification of H19 as a potential upstream lncRNA of miR-378a-5p and SERPINH1 in GC. **A** The lncRNA-miR-378a-5p regulatory network constructed by Cytoscape software. **B** The expression association of predicted lncRNAs with miR-378a-5p and SERPINH1 in GC. **C** The prognostic value of H19 in GC assessed by Kaplan-Meier plotter. **D** The expression boxplot of H19 in GC and normal samples determined by TCGA database. **E** The expression pattern between H19 and miR-378a-5p assessed by Spearman correlation. **F** The expression pattern between H19 and SERPINH1 assessed by Spearman correlation

Previous study has indicated that lncRNA can function as sponge to competitively bind to miRNA [26]. Thus, we used the LncBase v2 database to screen candidate upstream lncRNA that could potentially bind to hsa-miR-378a-5p. As shown in Fig. 6A, a total of 129 possible lncRNAs were identified. According to the ceRNA theory, the upstream lncRNA should be negatively associated with miRNA and meanwhile positively associated with mRNA. Therefore, we validate the expression pattern of those predicted lncRNAs based on TCGA project. Among all the 129 lncRNAs, seven lncRNAs were negatively associated with hsa-miR-378a-5p expression in GC, and only three of which (H19, PCOLCE-AS1, and INHBA-AS1) were positively associated with SERPINH1 and meanwhile overexpressed in GC (Fig. 6B). Then, survival analysis of these three lncRNAs showed that only H19 was significantly correlated with poor prognosis in GC patients (Fig. 6C). The expression boxplot of H19 in GC was presented in Fig. 6D, and its correlation with hsa-miR-378a-5p and SERPINH1 was presented in Fig. 6 E and F, respectively. Taken all these results into consideration, H19 serves as a candidate upstream lncRNA that could regulate hsa-miR-378a-5p, and H19/miR-378a-5p/SERPINH1 axis might be a potential regulatory pathway in GC.

### Profile of immune infiltration in GC

**Fig. 7:**
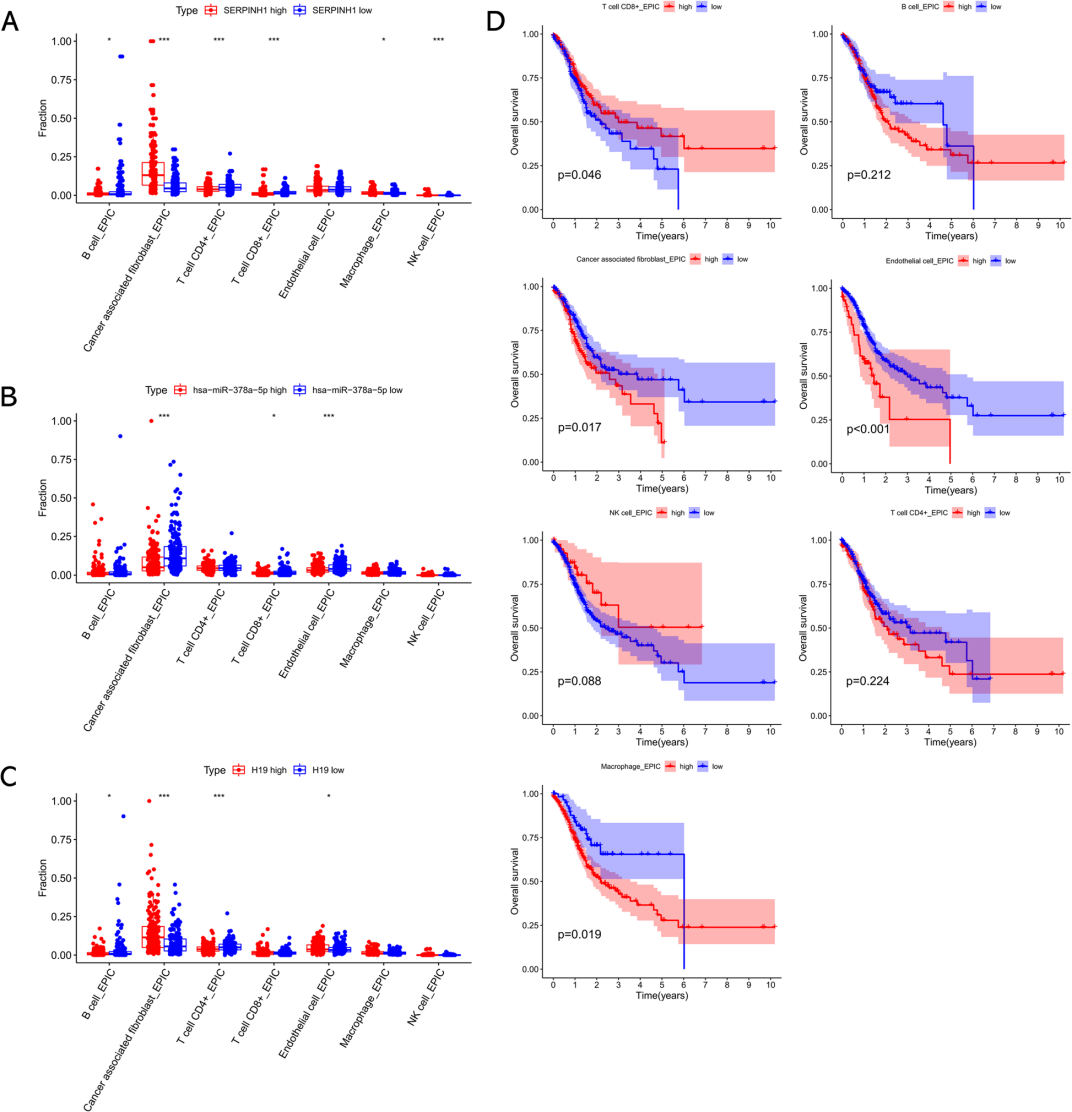
Immune cell infiltration analysis. **A** Boxplot for the different proportions of infiltrated immune cells between low- and high-SERPINH1 groups. **B** Boxplot for the different proportions of infiltrated immune cells between low- and high-miR-378a-5p groups. **C** Boxplot for the different proportions of infiltrated immune cells between low- and high-H19 groups. **D** The prognostic values of distinct immune cells in GC assessed by Kaplan-Meier plotter

We further evaluated the correlation of candidate biomarkers’ expression with immune cell infiltration level through the EPIC algorithm. As shown in Fig. 7A, infiltrating levels of cancer-associated fibroblasts (CAF) and macrophages were elevated in high SERPINH1 expression samples, whereas infiltrating levels of B cells, CD4+ T cells, and CD8+ T cells were downregulated in high SERPINH1 expression samples compared to low SERPINH1 expression samples. The infiltrating levels of CAFs, CD8+ T cells, and endothelial cells were decreased in high hsa-miR-378a-5p expression samples compared to low hsa-miR-378a-5p expression samples (Fig. 7B). In addition, GC patients with high H19 expression infiltrated with higher CAFs and endothelial cells and lower B cells and CD4+ T cells compared to those with low H19 expression (Fig. 7C). Survival analysis revealed that infiltrating levels of CD8+ T cells, CAFs, endothelial cells, and macrophages were significantly correlated with the prognosis of GC patients (Fig. 7D). All these findings suggested that H19 might regulate the immune cell infiltration in carcinogenesis of GC through miR-378a-5p/SERPINH1 signaling.

## Discussion

To date, the rapidly developed high-throughput sequencing technology and bioinformatics provide us with a more convenient platform to explore the pathogenesis of tumors at the gene level. In the present study, we successfully constructed a ceRNA network comprised of lncRNA (H19), miRNA (hsa-miR-378a-5p), and mRNA (SERPINH1) to provide a more comprehensive view of the RNA regulatory mechanism during GC carcinogenesis by combining multiple bioinformatic platforms.

We first identified three diagnostic biomarkers (FAP, PSAPL1, and SERPINH1) in GC by applying machine learning based on GEO and TCGA projects. Among them, FAP is a type 2 membrane-bound glycoprotein, which belongs to the serine protease family and has been identified as a marker of reactive tumor stromal fibroblasts [27]. FAP was reported to be highly expressed in GC tissues compared to normal controls, and patients in advanced pathological stage showed higher FAP expression levels than those in early pathological stage [28]. In addition, studies also found that FAP was overexpressed in GC cells, and FAP knocking down significantly restrained invasion and migration of GC cells by suppressing the activity of CAF [29]. SERPINH1, also known as HSP47, is an important collagen-specific molecule which is essential for the correct folding and secretion of distinct collagen types [30]. The mRNA and protein expression levels of SERPINH1 have been reported to be significantly upregulated in GC tissues compared with normal tissues, and inhibition of SERPINH1 significantly suppressed cancer cell migration and invasive abilities [31, 32]. Besides, upregulated SERPINH1 levels have been reported to be associated with poor prognosis in GC patients [32]. Studies focused on the oncogenic role of PSAPL1 in GC are limited to date, so they are worthy of future research.

MiRNAs are single-stranded noncoding RNAs that regulate gene expression through transcript degradation or inhibition of protein translation at posttranscriptional level [33]. Accumulating studies have indicated that miRNAs play vital roles in diverse biological processes of multiple diseases, including tumors [34, 35]. In the present study, the upstream miRNAs were predicted and validated based on bioinformatic database for the purpose of exploring candidate ceRNA regulating the diagnostic biomarkers mentioned above. As a result, hsa-miR-378a-5p was identified as a regulatory miRNA that could bind to SERPINH1 in GC. Studies have reported that the expression level of hsa-miR-378a-5p was downregulated in colorectal cancer (CRC) tissues compared to normal controls, and decreased hsa-miR-378a-5p level was significantly associated with advanced histological grade and worse prognosis in CRC patients [36]. Li et al. also indicated that hsa-miR-378a-5p serves as a tumor suppressive role in CRC, and overexpression of hsa-miR-378a-5p inhibited CRC cell proliferation by targeting CDK1. In addition, hsa-miR-378a-5p was reported to promote apoptosis of triple-negative breast cancer cells by targeting SUFU [37]. These results partially enhance the credibility of our finding that hsa-miR-378a-5p serves as a tumor suppressor in GC through targeting SERPINH1.

LncRNAs are a series of transcripts with length greater than 200 nucleotides and no protein-coding ability [38]. Similarly, the aberrant expression of lncRNA has been widely reported to participate in carcinogenesis and progression of diverse tumors [39, 40]. LncRNA H19 is a maternally expressed gene located at human chromosomal 11p15.5, which plays an important role in distinct pathologic processes [41, 42]. Accumulating studies showed that H19 serves as an oncogenic role and was upregulated in many malignancies, including breast cancer [42], ovarian cancer [43], lung cancer [44], and pancreatic cancer [45]. Moreover, existing evidences also demonstrated that H19 participates in GC progression through diverse pathways. For example, the expression level of H19 was found to be significantly upregulated in GC tissues and cell lines compared to that in the normal controls, and elevated H19 expression was remarkably related to advanced pathological stage in GC [46]. Besides, H19 serves as a prognostic biomarker in GC, and patients with high H19 expression showed a worse prognosis than those with low H19 expression [47]. H19 was also found to promote the epithelial-mesenchymal transition (EMT) and metastasis in GC by activating Wnt signaling [48]. Gan et al. indicated that H19 overexpression significantly enhanced, whereas H19 silencing suppressed the proliferation, migration, and invasion of GC cells through regulating miR-22-3p/Snail1 axis in vitro and in vivo [49]. In the present study, we found that H19 was overexpressed in GC and significantly associated with patients’ prognosis. Importantly, the expression pattern of H19 was negatively correlated with miR-378a-5p and meanwhile positively correlated with SERPINH1 in GC. Based on the ceRNA hypothesis, H19 was identified as the upstream regulatory lncRNA that could regulate the miR-378a-5p/SERPINH1 axis in GC.

In recent years, anticancer immunotherapy based on the reactivation of the host immunoreaction has revolutionized the treatment of patients with cancer and gained unprecedented progress [50]. However, the clinical use of immunotherapeutic agents is very limited due to the mechanisms of immune dysfunction in GC remain largely unclear. Emerging evidences have demonstrated that the interreaction between cancer cells and immune components in the tumor microenvironment (TME) is the determinant for tumor progression/regression. Thus, we further explored the correlation between the H19/miR-378a-5p/SERPINH1 axis and diverse TIICs in GC. In consequence, dysregulation of H19/miR-378a-5p/SERPINH1 axis was significantly correlated with altered infiltration abundances of CAFs, macrophages, B cells, CD4+ T cells, and CD8+ T cells in GC. CAFs are the prominent component of the tumor stroma, which supports the tumor cells by modifying the TME, boosting angiogenesis, and maintaining inflammatory status [51]. High infiltration of CAFs in TME could promote the malignant progression of GC [52]. Macrophages are the largest fraction of TIICs in the TME and can be divided into two major distinct subtypes according to their phenotype and function [53]. M1 macrophages are involved in the control of tumor growth by secreting pro-inflammatory cytokines, whereas M2 macrophages contribute to tumor progression by the production of immunosuppressive factors and chemokines [54]. B cells are pluripotent lineages which serve as antibody secreting cells but also serve as antigen-presenting cells (APCs) and immunoregulatory cells [55]. Studies have indicated that B-cell infiltration is associated with controlling tumor development in GC [56]. T cells exhibit important antitumor activities, and numerous studies showed that high proportions of infiltrating CD4+ and CD8+ T cells correlated with better prognosis in GC patients [57]. Interestingly, we found that CAFs were highly infiltrated in high H19 and high SERPINH1 groups, whereas its infiltration level in high miR-378a-5p was significantly downregulated. These findings imply that H19 might regulate the infiltration of CAFs to facilitate the carcinogenesis and progression of GC through miR-378a-5p/SERPINH1 pathway.

The present study inevitably exists a limitation that all results and conclusions were achieved based on online public databases; further experimental studies should be performed to validate our findings. Nevertheless, previous studies focused on constructing survival-related ceRNA network in GC are rare, and the present study successfully constructed a novel ceRNA regulatory network significantly associated with the prognosis of GC patients. Importantly, we proposed a novel hypothesis that H19 regulates the infiltration of CAFs to facilitate the carcinogenesis and progression of GC through miR-378a-5p/SERPINH1 signaling, which provides a promising clue for future research.

## Conclusion

In summary, based on machine learning and bioinformatics, the present study identified an immune-related prognostic ceRNA regulatory pathway that H19 might regulate the immune cell infiltration in carcinogenesis of GC through miR-378a-5p/SERPINH1 signaling.

## Supplementary Information

**Additional file 1: Supplementary Table 1**. DEGs between GC and normal samples.

## Data Availability

Thedatasets generated and/or analyzed during the current study are available in the Gene Expression Omnibus (GEO, https://www.ncbi.nlm.nih.gov/geo/) and The Cancer Genome Atlas (TCGA, https://www.cancer.gov/tcga) projects.

## Abbreviations

APC: Antigen-presenting cell
AUC: Area under receiver operating characteristic curve
BP: Biological processes
CAF: Cancer-associated fibroblasts
CC: Cellular components
ceRNA: Competing endogenous RNA
CRC: Colorectal cancer
DEG: Differentially expressed gene
DO: Disease ontology
EMT: Epithelial-mesenchymal transition
GC: Gastric cancer
GEO: Gene Expression Omnibus
GO: Gene ontology
KEGG: Kyoto Encyclopedia of Genes and Genomes
LASSO: Least absolute shrinkage and selection operator
lncRNA: Long noncoding RNA
MF: Molecular functions
miRNA: Micro RNA
ncRNA: Noncoding RNA
SVA: Surrogate variable analysis
SVM-RFE: Support vector machine recursive feature elimination
TCGA: The Cancer Genome Atlas
TIIC: Tumor-infiltrating immune cell
TME: Tumor microenvironment
