# Supplementary material for: H19 may regulate the immune cell infiltration in carcinogenesis of gastric cancer through miR-378a-5p/SERPINH1 signaling
Source: World J Surg Oncol. 2022 Sep 14;20:295. doi: 10.1186/s12957-022-02760-6 (PMC9472414; doi:10.1186/s12957-022-02760-6)
Supplement: Supplementary file 1 — Additional file 1: Supplementary Table 1. DEGs between GC and normal samples. [file 12957_2022_2760_MOESM1_ESM.docx]

| Supplementary Table 1. DEGs between GC and normal samples | | | | | | |
| --- | --- | --- | --- | --- | --- | --- |
| id | logFC | AveExpr | t | *P*.Value | adj.*P*.Val | B |
| ATP4A | -6.5701 | 9.405952 | -12.9958 | 1.94E-24 | 3.13E-21 | 44.98215 |
| GIF | -6.53236 | 10.95672 | -12.3597 | 5.91E-23 | 5.41E-20 | 41.63069 |
| ATP4B | -5.87151 | 8.926338 | -13.3419 | 3.07E-25 | 8.30E-22 | 46.7923 |
| GKN1 | -5.7466 | 12.08994 | -9.13836 | 2.34E-15 | 1.47E-13 | 24.44692 |
| LIPF | -5.4045 | 12.37159 | -9.85596 | 4.80E-17 | 4.91E-15 | 28.26405 |
| GKN2 | -5.39735 | 12.381 | -10.3717 | 2.90E-18 | 4.42E-16 | 31.02241 |
| KCNE2 | -5.05452 | 9.94285 | -11.1905 | 3.35E-20 | 1.13E-17 | 35.40533 |
| SOSTDC1 | -4.87079 | 9.238404 | -10.8198 | 2.52E-19 | 5.75E-17 | 33.42168 |
| CHIA | -4.65696 | 7.932882 | -9.43758 | 4.65E-16 | 3.58E-14 | 26.03444 |
| ESRRG | -4.46842 | 9.385193 | -12.0774 | 2.71E-22 | 1.84E-19 | 40.13405 |
| LTF | -4.29998 | 10.92669 | -7.88614 | 1.83E-12 | 5.12E-11 | 17.91364 |
| CHGA | -4.04319 | 9.697123 | -10.8058 | 2.72E-19 | 6.01E-17 | 33.34671 |
| PGC | -3.97426 | 13.2494 | -8.24426 | 2.78E-13 | 9.67E-12 | 19.75884 |
| KCNJ16 | -3.94923 | 7.911813 | -12.4928 | 2.89E-23 | 3.29E-20 | 42.33413 |
| PSCA | -3.94563 | 11.40894 | -9.0144 | 4.57E-15 | 2.62E-13 | 23.7915 |
| CWH43 | -3.92377 | 6.529358 | -13.5541 | 9.95E-26 | 4.31E-22 | 47.89627 |
| AQP4 | -3.91411 | 6.716108 | -11.2435 | 2.51E-20 | 8.62E-18 | 35.68917 |
| DPCR1 | -3.87433 | 10.7116 | -10.21 | 7.00E-18 | 9.15E-16 | 30.15666 |
| SCGB2A1 | -3.84354 | 8.100883 | -9.10295 | 2.83E-15 | 1.73E-13 | 24.25957 |
| FUT9 | -3.82875 | 8.14737 | -10.5532 | 1.08E-18 | 1.93E-16 | 31.99423 |
| C16orf89 | -3.76836 | 8.708086 | -10.4021 | 2.46E-18 | 3.83E-16 | 31.18487 |
| SST | -3.75485 | 9.730506 | -9.04984 | 3.77E-15 | 2.21E-13 | 23.97874 |
| CXCL17 | -3.74925 | 11.19989 | -8.91861 | 7.64E-15 | 4.09E-13 | 23.28601 |
| LINC00261 | -3.73685 | 9.608489 | -8.87014 | 9.91E-15 | 5.13E-13 | 23.03063 |
| ETNPPL | -3.66488 | 7.304629 | -9.24871 | 1.29E-15 | 8.71E-14 | 25.03157 |
| SH3GL2 | -3.65654 | 6.179321 | -10.9073 | 1.56E-19 | 4.00E-17 | 33.89005 |
| VSIG1 | -3.61845 | 10.83781 | -10.0193 | 1.98E-17 | 2.28E-15 | 29.13699 |
| MFSD4 | -3.60047 | 9.270568 | -11.3688 | 1.27E-20 | 4.73E-18 | 36.35852 |
| TMED6 | -3.52622 | 8.691431 | -8.68712 | 2.64E-14 | 1.21E-12 | 22.06861 |
| FBP2 | -3.44584 | 6.312731 | -10.2096 | 7.01E-18 | 9.15E-16 | 30.15464 |
| PDILT | -3.43741 | 6.272786 | -9.03377 | 4.11E-15 | 2.39E-13 | 23.89383 |
| CPA2 | -3.37341 | 9.766259 | -10.9067 | 1.57E-19 | 4.00E-17 | 33.8871 |
| MAP7D2 | -3.34254 | 7.671925 | -9.70349 | 1.10E-16 | 9.84E-15 | 27.4504 |
| ANXA10 | -3.28129 | 12.61525 | -8.12137 | 5.32E-13 | 1.73E-11 | 19.12313 |
| KRT20 | -3.26056 | 11.45778 | -7.51015 | 1.29E-11 | 2.88E-10 | 16.00298 |
| MSMB | -3.25602 | 9.657125 | -5.52375 | 2.04E-07 | 1.66E-06 | 6.581998 |
| FAM3B | -3.20796 | 11.01204 | -7.49536 | 1.39E-11 | 3.06E-10 | 15.92843 |
| SLC26A9 | -3.19547 | 9.051914 | -10.5372 | 1.18E-18 | 2.09E-16 | 31.9083 |
| MUC5AC | -3.18995 | 11.47535 | -9.67297 | 1.30E-16 | 1.13E-14 | 27.28771 |
| GC | -3.18598 | 8.031787 | -8.01691 | 9.22E-13 | 2.84E-11 | 18.5848 |
| VSIG2 | -3.1711 | 10.2823 | -11.4029 | 1.05E-20 | 4.15E-18 | 36.54074 |
| TFF2 | -3.16183 | 13.16112 | -7.12997 | 8.97E-11 | 1.60E-09 | 14.10387 |
| MT1M | -3.16174 | 9.871185 | -10.6053 | 8.12E-19 | 1.58E-16 | 32.27291 |
| AKR1B10 | -3.15978 | 13.08124 | -9.97645 | 2.49E-17 | 2.76E-15 | 28.90771 |
| ADH1C | -3.11327 | 11.36086 | -7.83652 | 2.37E-12 | 6.41E-11 | 17.65982 |
| ALDH3A1 | -3.08579 | 9.8315 | -10.0041 | 2.15E-17 | 2.45E-15 | 29.05563 |
| C6orf58 | -3.07734 | 9.082017 | -6.12377 | 1.26E-08 | 1.36E-07 | 9.280827 |
| CLIC6 | -3.03956 | 10.11108 | -10.2177 | 6.71E-18 | 8.91E-16 | 30.19766 |
| CKM | -3.01659 | 5.90466 | -9.17821 | 1.89E-15 | 1.21E-13 | 24.65796 |
| RDH12 | -3.0003 | 7.663794 | -11.6713 | 2.45E-21 | 1.23E-18 | 37.97318 |
| LOC101926959 | -2.99149 | 5.856838 | -8.94754 | 6.54E-15 | 3.57E-13 | 23.43857 |
| MAL | -2.99139 | 9.129489 | -13.3163 | 3.51E-25 | 8.46E-22 | 46.65887 |
| DNER | -2.94455 | 7.609273 | -9.75481 | 8.32E-17 | 7.85E-15 | 27.72418 |
| PSAPL1 | -2.93913 | 9.563403 | -14.653 | 3.12E-28 | 2.26E-24 | 53.54205 |
| APLP1 | -2.9192 | 6.536588 | -9.16632 | 2.01E-15 | 1.29E-13 | 24.59498 |
| CAPN9 | -2.88728 | 8.998444 | -12.3152 | 7.51E-23 | 6.51E-20 | 41.39508 |
| PTPRZ1 | -2.84546 | 8.672381 | -8.80468 | 1.41E-14 | 7.03E-13 | 22.68609 |
| GHRL | -2.83929 | 8.660022 | -11.1139 | 5.08E-20 | 1.64E-17 | 34.99563 |
| SCNN1B | -2.82217 | 6.982975 | -10.7167 | 4.42E-19 | 9.21E-17 | 32.86951 |
| KLK11 | -2.80651 | 9.34555 | -8.31215 | 1.94E-13 | 6.98E-12 | 20.11107 |
| CLDN18 | -2.77535 | 12.24494 | -7.63814 | 6.65E-12 | 1.62E-10 | 16.65001 |
| CCKBR | -2.75691 | 7.782329 | -11.5624 | 4.43E-21 | 2.00E-18 | 37.3922 |
| AZGP1 | -2.74144 | 10.25342 | -9.42963 | 4.85E-16 | 3.73E-14 | 25.9922 |
| MUC6 | -2.7342 | 10.20369 | -7.28699 | 4.04E-11 | 7.88E-10 | 14.88387 |
| HPGD | -2.73404 | 11.15097 | -10.5267 | 1.25E-18 | 2.14E-16 | 31.85213 |
| CKMT2 | -2.69764 | 8.230741 | -10.5576 | 1.05E-18 | 1.90E-16 | 32.01785 |
| RFX6 | -2.68597 | 6.53675 | -8.21914 | 3.18E-13 | 1.09E-11 | 19.62869 |
| GUCA2B | -2.67605 | 6.175491 | -8.22274 | 3.12E-13 | 1.07E-11 | 19.64733 |
| GPR64 | -2.65285 | 8.345391 | -9.82526 | 5.68E-17 | 5.72E-15 | 28.10015 |
| C11orf92 | -2.64756 | 8.523902 | -9.42361 | 5.01E-16 | 3.84E-14 | 25.96018 |
| TRIM50 | -2.64262 | 6.624301 | -11.0065 | 9.12E-20 | 2.60E-17 | 34.42095 |
| DUOX2 | -2.62543 | 10.38639 | -7.33889 | 3.10E-11 | 6.27E-10 | 15.14305 |
| PIK3C2G | -2.62209 | 9.046597 | -8.48979 | 7.57E-14 | 3.08E-12 | 21.03602 |
| REG1A | -2.61822 | 13.02682 | -5.89316 | 3.74E-08 | 3.59E-07 | 8.225721 |
| FCGBP | -2.61376 | 11.06014 | -7.63781 | 6.66E-12 | 1.62E-10 | 16.64831 |
| GPR155 | -2.60474 | 9.374814 | -10.3033 | 4.21E-18 | 5.96E-16 | 30.65585 |
| RP11-363E7.4 | -2.58295 | 9.952188 | -10.4146 | 2.29E-18 | 3.64E-16 | 31.25212 |
| SYTL5 | -2.5782 | 8.19589 | -9.66064 | 1.39E-16 | 1.20E-14 | 27.22197 |
| REG3A | -2.56779 | 10.26296 | -5.69698 | 9.27E-08 | 8.15E-07 | 7.345373 |
| GATA6-AS1 | -2.55529 | 10.06255 | -13.0252 | 1.66E-24 | 3.00E-21 | 45.13628 |
| ADH7 | -2.54813 | 7.518778 | -11.7094 | 1.99E-21 | 1.05E-18 | 38.17604 |
| CA9 | -2.53811 | 10.27505 | -9.76827 | 7.74E-17 | 7.41E-15 | 27.79598 |
| CNTN3 | -2.53117 | 6.641802 | -8.96338 | 6.01E-15 | 3.31E-13 | 23.52214 |
| CA2 | -2.50741 | 13.10327 | -8.99266 | 5.13E-15 | 2.88E-13 | 23.67668 |
| LOC400043 | -2.49672 | 10.61615 | -9.73168 | 9.44E-17 | 8.66E-15 | 27.60078 |
| SSTR1 | -2.48845 | 8.437075 | -10.5614 | 1.03E-18 | 1.88E-16 | 32.03792 |
| CNTD1 | -2.44706 | 7.075724 | -8.01136 | 9.49E-13 | 2.92E-11 | 18.55623 |
| LINC00982 | -2.43871 | 7.734932 | -11.406 | 1.04E-20 | 4.15E-18 | 36.55734 |
| FAM150B | -2.43807 | 7.323191 | -9.56911 | 2.28E-16 | 1.86E-14 | 26.73433 |
| DUOXA2 | -2.41188 | 8.137863 | -6.98963 | 1.82E-10 | 3.04E-09 | 13.41235 |
| IRX3 | -2.3935 | 9.047664 | -8.70643 | 2.38E-14 | 1.11E-12 | 22.16996 |
| TNFRSF17 | -2.39061 | 9.209547 | -7.25696 | 4.71E-11 | 9.06E-10 | 14.73421 |
| PDIA2 | -2.37358 | 6.549465 | -9.52349 | 2.92E-16 | 2.32E-14 | 26.49144 |
| PLCXD3 | -2.36537 | 6.747853 | -9.75164 | 8.47E-17 | 7.94E-15 | 27.70725 |
| GSTA3 | -2.365 | 6.5988 | -7.68641 | 5.17E-12 | 1.30E-10 | 16.89496 |
| KIAA1324 | -2.35312 | 10.2476 | -7.75702 | 3.59E-12 | 9.31E-11 | 17.25414 |
| CAPN13 | -2.31564 | 7.661345 | -11.8072 | 1.17E-21 | 7.05E-19 | 38.69705 |
| CYP2C18 | -2.31348 | 10.0944 | -9.73594 | 9.22E-17 | 8.50E-15 | 27.6235 |
| ADTRP | -2.29856 | 10.27834 | -11.847 | 9.45E-22 | 5.85E-19 | 38.90882 |
| GSTA1 | -2.28944 | 10.39448 | -7.91428 | 1.58E-12 | 4.49E-11 | 18.0578 |
| LYPD6B | -2.28024 | 9.598343 | -10.8197 | 2.52E-19 | 5.75E-17 | 33.42143 |
| KCNJ15 | -2.27841 | 8.299576 | -11.0561 | 6.95E-20 | 2.06E-17 | 34.68674 |
| UGT2B15 | -2.27518 | 9.238242 | -8.51376 | 6.66E-14 | 2.74E-12 | 21.16118 |
| TCN1 | -2.2691 | 11.52208 | -6.35583 | 4.15E-09 | 4.97E-08 | 10.3631 |
| SIGLEC11 | -2.26505 | 7.290502 | -9.40495 | 5.55E-16 | 4.20E-14 | 25.86099 |
| SMIM5 | -2.26037 | 8.758689 | -12.3569 | 6.00E-23 | 5.41E-20 | 41.61567 |
| BPIFB1 | -2.25247 | 10.35317 | -3.39656 | 0.000933 | 0.003202 | -1.45483 |
| PKIB | -2.23201 | 9.182564 | -10.0877 | 1.36E-17 | 1.64E-15 | 29.50253 |
| SULT2A1 | -2.2312 | 7.160762 | -6.62219 | 1.13E-09 | 1.54E-08 | 11.62889 |
| SLC16A7 | -2.23024 | 8.555908 | -10.3158 | 3.93E-18 | 5.67E-16 | 30.72309 |
| KCNJ13 | -2.1983 | 5.532074 | -9.00317 | 4.85E-15 | 2.74E-13 | 23.73217 |
| AADAC | -2.18851 | 10.00023 | -7.22739 | 5.47E-11 | 1.03E-09 | 14.58704 |
| SULT1C2 | -2.18786 | 11.14193 | -8.69212 | 2.57E-14 | 1.19E-12 | 22.09486 |
| CTSE | -2.18359 | 13.33726 | -7.5029 | 1.34E-11 | 2.97E-10 | 15.96644 |
| PNLIPRP2 | -2.17862 | 7.474629 | -5.84272 | 4.73E-08 | 4.45E-07 | 7.997846 |
| GATA5 | -2.17406 | 6.804944 | -8.4943 | 7.39E-14 | 3.02E-12 | 21.05957 |
| LIFR | -2.16643 | 8.7284 | -11.186 | 3.43E-20 | 1.14E-17 | 35.38132 |
| SPINK2 | -2.1533 | 6.67268 | -8.24733 | 2.74E-13 | 9.56E-12 | 19.77473 |
| SMIM24 | -2.15303 | 10.99176 | -7.63562 | 6.73E-12 | 1.63E-10 | 16.63722 |
| ARL14 | -2.15065 | 10.59993 | -7.75649 | 3.60E-12 | 9.32E-11 | 17.25146 |
| IGFBP2 | -2.14548 | 11.4857 | -7.77326 | 3.30E-12 | 8.66E-11 | 17.3369 |
| PP7080 | -2.14269 | 9.833908 | -9.91591 | 3.47E-17 | 3.64E-15 | 28.58424 |
| SCNN1G | -2.13802 | 6.714154 | -9.9659 | 2.64E-17 | 2.90E-15 | 28.85133 |
| ZNF385B | -2.12165 | 6.834477 | -8.31998 | 1.86E-13 | 6.74E-12 | 20.15176 |
| EYA2 | -2.10086 | 7.774838 | -7.09477 | 1.07E-10 | 1.87E-09 | 13.9299 |
| TFF1 | -2.09144 | 14.22133 | -6.71562 | 7.14E-10 | 1.02E-08 | 12.07844 |
| GPER1 | -2.08896 | 6.271118 | -7.65483 | 6.10E-12 | 1.49E-10 | 16.73465 |
| AKR1C1 | -2.07343 | 10.6212 | -9.23128 | 1.42E-15 | 9.42E-14 | 24.93915 |
| CPB1 | -2.07287 | 6.556656 | -6.84832 | 3.69E-10 | 5.69E-09 | 12.72163 |
| HEPACAM2 | -2.06334 | 7.968164 | -6.67537 | 8.71E-10 | 1.22E-08 | 11.88443 |
| NKX2-3 | -2.06129 | 6.657445 | -8.47065 | 8.38E-14 | 3.36E-12 | 20.93615 |
| CAPN8 | -2.06082 | 11.12403 | -9.253 | 1.26E-15 | 8.56E-14 | 25.05429 |
| FOLR1 | -2.06028 | 9.516849 | -8.83332 | 1.21E-14 | 6.15E-13 | 22.83678 |
| TPH1 | -2.03915 | 6.461077 | -8.88962 | 8.93E-15 | 4.67E-13 | 23.13326 |
| PAIP2B | -2.03902 | 8.662474 | -8.49024 | 7.55E-14 | 3.08E-12 | 21.03835 |
| ERO1LB | -2.03897 | 8.624687 | -9.57614 | 2.19E-16 | 1.81E-14 | 26.77174 |
| RNASE1 | -2.03784 | 11.26929 | -10.9989 | 9.50E-20 | 2.64E-17 | 34.38068 |
| MT1G | -2.02493 | 11.24609 | -9.798 | 6.58E-17 | 6.45E-15 | 27.95463 |
| SCARA5 | -2.01841 | 7.861014 | -9.11183 | 2.70E-15 | 1.66E-13 | 24.30652 |
| PBLD | -2.01081 | 10.63564 | -11.5272 | 5.36E-21 | 2.32E-18 | 37.20435 |
| PMEPA1 | 2.012655 | 9.761666 | 11.38881 | 1.14E-20 | 4.40E-18 | 36.46556 |
| MMP3 | 2.029895 | 8.53441 | 6.339076 | 4.50E-09 | 5.35E-08 | 10.28431 |
| HMGB3P1 | 2.039967 | 7.282931 | 10.31284 | 4.00E-18 | 5.69E-16 | 30.70708 |
| CDH17 | 2.041522 | 9.678527 | 3.945154 | 0.000136 | 0.000585 | 0.347086 |
| CRISPLD1 | 2.075094 | 7.367269 | 9.710929 | 1.06E-16 | 9.49E-15 | 27.4901 |
| BYSL | 2.076867 | 7.943365 | 10.5311 | 1.22E-18 | 2.12E-16 | 31.87571 |
| CLDN2 | 2.079405 | 7.842845 | 7.380572 | 2.50E-11 | 5.20E-10 | 15.3517 |
| BGN | 2.084491 | 9.183604 | 11.27477 | 2.11E-20 | 7.51E-18 | 35.85615 |
| DUXAP10 | 2.103529 | 5.365589 | 6.669375 | 8.97E-10 | 1.26E-08 | 11.85558 |
| EFNA3 | 2.12027 | 6.570205 | 8.088729 | 6.32E-13 | 2.00E-11 | 18.95471 |
| WISP1 | 2.120929 | 6.642986 | 11.88345 | 7.76E-22 | 4.94E-19 | 39.10303 |
| CDCA5 | 2.131318 | 7.740727 | 9.647676 | 1.49E-16 | 1.27E-14 | 27.15287 |
| HOXA13 | 2.140868 | 7.006348 | 8.814718 | 1.33E-14 | 6.72E-13 | 22.73891 |
| HCAR3 | 2.173295 | 6.675471 | 5.63692 | 1.22E-07 | 1.04E-06 | 7.079183 |
| CLRN3 | 2.192439 | 8.702058 | 4.764036 | 5.50E-06 | 3.26E-05 | 3.406043 |
| PLA2G2A | 2.196048 | 9.973286 | 5.289 | 5.81E-07 | 4.27E-06 | 5.569602 |
| RP3-428L16.2 | 2.208652 | 6.557123 | 8.750395 | 1.88E-14 | 9.02E-13 | 22.40077 |
| FOXM1 | 2.214879 | 7.648345 | 7.89139 | 1.78E-12 | 5.00E-11 | 17.94051 |
| RARRES1 | 2.228247 | 8.669903 | 8.896087 | 8.62E-15 | 4.53E-13 | 23.16732 |
| EPHX4 | 2.258813 | 5.886111 | 8.062788 | 7.24E-13 | 2.27E-11 | 18.82099 |
| CTHRC1 | 2.280613 | 10.76203 | 10.66262 | 5.94E-19 | 1.20E-16 | 32.58001 |
| SFRP4 | 2.292517 | 8.265643 | 9.017478 | 4.49E-15 | 2.59E-13 | 23.80776 |
| S100A2 | 2.332617 | 7.417242 | 7.463144 | 1.64E-11 | 3.55E-10 | 15.76625 |
| KRT80 | 2.332754 | 5.714235 | 8.778965 | 1.62E-14 | 7.90E-13 | 22.5509 |
| HOXC6 | 2.363794 | 8.02431 | 9.255097 | 1.25E-15 | 8.52E-14 | 25.06544 |
| LY6E | 2.413995 | 9.098761 | 9.988118 | 2.34E-17 | 2.60E-15 | 28.97008 |
| CLDN3 | 2.421769 | 8.0921 | 6.098858 | 1.42E-08 | 1.51E-07 | 9.165817 |
| MFAP2 | 2.437535 | 8.477804 | 12.14721 | 1.86E-22 | 1.39E-19 | 40.50455 |
| CXCL8 | 2.468982 | 9.818517 | 7.763423 | 3.47E-12 | 9.08E-11 | 17.28679 |
| SULF1 | 2.514205 | 9.780276 | 12.4396 | 3.84E-23 | 4.16E-20 | 42.05323 |
| FKBP10 | 2.594013 | 6.479905 | 10.25824 | 5.38E-18 | 7.23E-16 | 30.41489 |
| CEMIP | 2.619328 | 6.445474 | 10.84358 | 2.21E-19 | 5.33E-17 | 33.54901 |
| CLDN1 | 2.634372 | 8.258419 | 11.95931 | 5.14E-22 | 3.37E-19 | 39.50652 |
| COL8A1 | 2.697135 | 7.531461 | 13.16981 | 7.67E-25 | 1.66E-21 | 45.89355 |
| MAGEA6 | 2.744278 | 6.542498 | 5.867743 | 4.21E-08 | 4.00E-07 | 8.110759 |
| THBS2 | 2.813622 | 9.582531 | 10.95096 | 1.23E-19 | 3.38E-17 | 34.1239 |
| CLDN7 | 2.87761 | 9.848674 | 8.005376 | 9.79E-13 | 2.99E-11 | 18.52546 |
| SPP1 | 2.962689 | 8.753062 | 10.50514 | 1.40E-18 | 2.37E-16 | 31.73669 |
| ZIC2 | 2.991268 | 5.871688 | 7.771997 | 3.32E-12 | 8.70E-11 | 17.33048 |
| HOXA10 | 3.120682 | 7.117365 | 10.5003 | 1.44E-18 | 2.38E-16 | 31.71079 |
| SERPINH1 | 3.243492 | 9.101352 | 15.55982 | 2.96E-30 | 6.41E-26 | 58.09913 |
| COL10A1 | 3.310026 | 7.587191 | 11.08422 | 5.97E-20 | 1.82E-17 | 34.83709 |
| FAP | 3.541492 | 7.302778 | 14.12776 | 4.84E-27 | 2.62E-23 | 50.85964 |
| COL11A1 | 3.613764 | 6.333701 | 9.645584 | 1.51E-16 | 1.28E-14 | 27.14173 |
| INHBA | 3.643111 | 7.595346 | 14.89071 | 9.13E-29 | 9.89E-25 | 54.74616 |
| CDH3 | 3.661927 | 7.182189 | 12.92712 | 2.80E-24 | 4.05E-21 | 44.62194 |
| FNDC1 | 3.905695 | 7.287048 | 11.51344 | 5.78E-21 | 2.45E-18 | 37.13107 |
| CST1 | 4.029613 | 6.761387 | 10.93109 | 1.37E-19 | 3.67E-17 | 34.01755 |
